# Extracting Information from Qubit-Environment Correlations

**DOI:** 10.1038/srep07443

**Published:** 2014-12-17

**Authors:** John H. Reina, Cristian E. Susa, Felipe F. Fanchini

**Affiliations:** 1 Departamento de Física, Universidad del Valle, A.A. 25360, Cali, Colombia; 2 Centre for Bioinformatics and Photonics—CIBioFI, Calle 13 No. 100-00, Edificio 320, No. 1069, Cali, Colombia; 3 Departamento de Física, Faculdade de Ciências, UNESP, Bauru, SP, CEP 17033-360, Brazil

## Abstract

Most works on open quantum systems generally focus on the reduced physical system by tracing out the environment degrees of freedom. Here we show that the qubit distributions with the environment are essential for a thorough analysis, and demonstrate that the way that quantum correlations are distributed in a quantum register is constrained by the way in which each subsystem gets correlated with the environment. For a two-qubit system coupled to a common dissipative environment 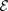, we show how to optimise interqubit correlations and entanglement via a quantification of the qubit-environment information flow, in a process that, perhaps surprisingly, does not rely on the knowledge of the state of the environment. To illustrate our findings, we consider an optically-driven bipartite interacting qubit *AB* system under the action of 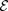. By tailoring the light-matter interaction, a relationship between the qubits early stage disentanglement and the qubit-environment entanglement distribution is found. We also show that, under suitable initial conditions, the qubits energy asymmetry allows the identification of physical scenarios whereby qubit-qubit entanglement minima coincide with the extrema of the 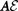 and 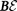 entanglement oscillations.

The quantum properties of physical systems have been studied for many years as crucial resources for quantum processing tasks and quantum information protocols^1^,^2^,^3^,^4^,^5^. Among these properties, entanglement, non-locality, and correlations between quantum objects arise as fundamental features^6^,^7^. The study of such properties in open quantum systems is a crucial aspect of quantum information science^8^,^9^, in particular because decoherence appears as a ubiquituous physical process that prevents the realisation of unitary quantum dynamics—it washes out quantum coherence and multipartite correlation effects, and it has long been recognised as a mechanism responsible for the emergence of classicality from events in a purely quantum realm^10^. In fact, it is the influence of harmful errors caused by the interaction of a quantum register with its environment^10^,^11^,^12^,^13^ that precludes the construction of an efficient scalable quantum computer^14^,^15^.

Many works devoted to the study of entanglement and correlations dynamics in open quantum systems are focused on the analysis of the reduced system of interest (the register) and the quantum state of the environment is usually discarded^16^,^17^,^18^,^19^,^20^. There have recently been proposed, however, some ideas for detecting system-environment correlations (see e.g., refs. 21, 22, and references therein). For example, experimental tests of system-environment correlations detection have been recently carried out by means of single trapped ions^23^. The role and effect of the system-environment correlations on the dynamics of open quantum systems have also been studied within the spin-boson model^24^,^25^, and as a precursor of a non-Markovian dynamics^26^. Here, we approach the qubit-environment dynamics from a different perspective and show that valuable information about the evolution of quantum entanglement and correlations can be obtained if the flow of information between the register and the environment is better understood.

It is a known fact that a quantum system composed by many parts cannot freely share entanglement or quantum correlations between its parts^27^,^28^,^29^,^30^,^31^,^32^. Indeed, there are strong constrains on how these correlations can be shared, which gives rise to what is known as monogamy of quantum correlations. In this paper we use monogamic relations to demonstrate that the way that quantum correlations are distributed in a quantum register is constrained by the way in which each subsystem gets correlated with the reservoir^27^,^33^,^34^, and that an optimisation of the interqubit entanglement and correlations can be devised via a quantification of the information flow between each qubit and its environment.

We consider a bipartite *AB* system (the qubits) interacting with a third subsystem 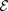 (the environment). We begin by assuming that the *whole* ‘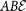 system’ is described by an initial pure state 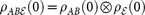; i.e., at *t* = 0, the qubits and the environment density matrices, *ρ_AB_* and 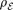, need to be pure.

The global 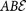 evolution is given by 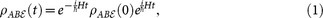
where *H* denotes the Hamiltonian of the tripartite system. Since 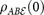 is pure, 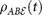 is also pure for all time and hence we can calculate the way that *AB* gets entangled with the environment directly from the entropy. For the entanglement of formation 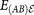, for example, this is given by the von Neumann entropy 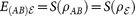
3,35. In order to quantify the way in which *A* (*B*) gets entangled with 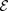, we calculate 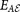 (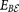) by means of the Koashi-Winter (KW) relations (see the Methods section)^27^,^34^

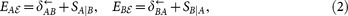
where 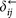 denotes the quantum discord^36^,^37^,^38^, and *S_i_*_|*j*_ is the conditional entropy^6^,^7^. Since the tripartite state 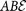 remains pure for all time *t*, we can calculate, even without any knowledge about 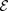, the entanglement *E_ij_* between each subsystem and the environment. We do so by means of discord. We also compute the quantum discord between each subsystem and the environment as (see the Methods section) 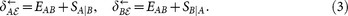
We note that, in general, 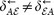 and 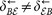, i.e., these quantities are not symmetric. Directly from the KW relations, such asymmetry can be understood due to the different behaviour exhibited by the entanglement of formation and the discord for the *AB* partition; e.g., 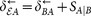, such that 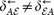 when 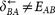 (the equality holds for bipartite pure states). In our setup the *AB* partition goes into a mixed state due to the dissipative effects and the qubits detuning^39^,^40^. In our calculations, the behaviour of 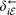 and 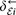, *i* = *A*, *B*, is similar, so we only compute, without loss of generality, those correlations given by equations (3). An important aspect to be emphasised on the KW relations concerns its definition in terms of the entanglement of formation. Although the original version of the KW relations is given in terms of the entanglement of formation and classical correlations defined in terms of the von Neumann entropy, this is not a necessary condition. Indeed, similar monogamic relations can be determined by any concave measure of entanglement. In this sense, we can define a KW relation in terms of the tangle or even the concurrence, since they both obey the concave property. For instance, in [41] the authors use the KW relation in terms of the linear entropy to show that the tangle is monogamous for a system of *N* qubits. Here we use the entanglement of formation and quantum discord given their nice operational interpretations, but we stress that this is not a necessary condition.

We illustrate the above statements by considering qubits that are represented by two-level quantum emitters, where |0*_i_*〉, and |1*_i_*〉 denote the ground and excited state of emitter *i*, respectively, with individual transition frequencies *ω_i_*, and in interaction with a common environment (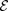) comprised by the vacuum quantised radiation field^39^,^40^, as schematically shown in Fig. 1(a), where 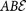 denotes the strength of the interaction between the qubits.

The total Hamiltonian describing the dynamics of the whole 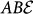 system can be written as 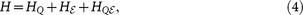
where the qubits free energy 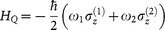, the environment Hamiltonian 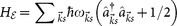, and the qubit-environment interaction, in the dipole approximation, 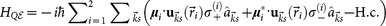, where 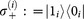, and 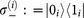 are the raising and lowering Pauli operators acting on the qubit *i*, 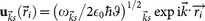 is the coupling constant, 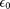 the vacuum permittivity, *ϑ* the quantisation volume, 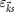 the unitary vector of the field mode, 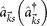 are the annihilation (creation) operators of the mode, and 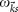 is its frequency.

For the sake of completeness, we also allow for an external qubit control whereby the qubits can be optically-driven by a coherent laser field of frequency *ω_L_*, 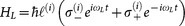, where ħ*ℓ_i_* = −***µ****_i_*·***E****_i_* gives the qubit-field coupling, with ***µ****_i_* being the *i*-th transition dipole moment and ***E****_i_* the amplitude of the coherent driving acting on qubit *i* located at position 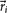. The two emitters are separated by the vector 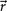 and are characterised by transition dipole moments ***µ****_i_*≡〈0*_i_*|**D***_i_*|1*_i_*〉, with dipole operators **D***_i_*, and spontaneous emission rates Γ*_i_*.

Given the features of the considered physical system, we may assume a weak system-environment coupling such that the Born-Markov approximation is valid, and we work within the rotating wave approximation for both the system-environment and the system-external laser Hamiltonians^42^. Within this framework, the effective Hamiltonian of the reduced two-qubit *AB* system, which takes into account both the effects of the interaction with the environment and the interaction with the coherent laser field, can be written as 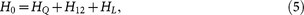
where 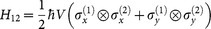, and *V* is the strength of the dipole-dipole (qubit) coupling which depends on the separation and orientation between the dipoles^39^,^40^,^42^.

In order to impose the pure initial condition to the 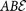 system required to use the KW relations, we suppose that the quantum register is in a pure initial state and that we have a zero temperature environment. Thus, 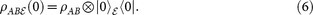
However, we note that a less controllable and different initial state for the environment can be considered since an appropriate purification of the environment 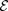 with a new subsystem 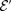 could be realised. Despite this, for the sake of simplicity in calculating the quantum register dynamics, we consider a zero temperature environment.

The results below reported require a quantification of the qubits dissipative dynamics. This is described by means of the quantum master equation^39^,^40^: 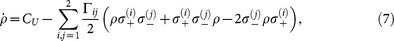
where the commutator 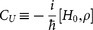 gives the unitary part of the evolution. The individual and collective spontaneous emission rates are considered such that Γ*_ii_* = Γ*_i_* ≡ Γ, and 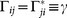, respectively. For simplicity of writing, we adopt the notation *ρ_ij_*, where *i*, *j* = 1, 2, 3, 4 for the 16 density matrix elements; Σ*_i_ρ_ii_* = 1.

The master equation (7) gives a solution for *ρ_AB_*(*t*) that becomes mixed since it creates quantum correlations with the environment. We pose the following questions: i) How does each qubit get entangled with the environment? ii) How does this depend on the energy mismatch between *A* and *B*?, and iii) on the external laser pumping?

## Results

### Quantum register-environment correlations

**Figure 1:**
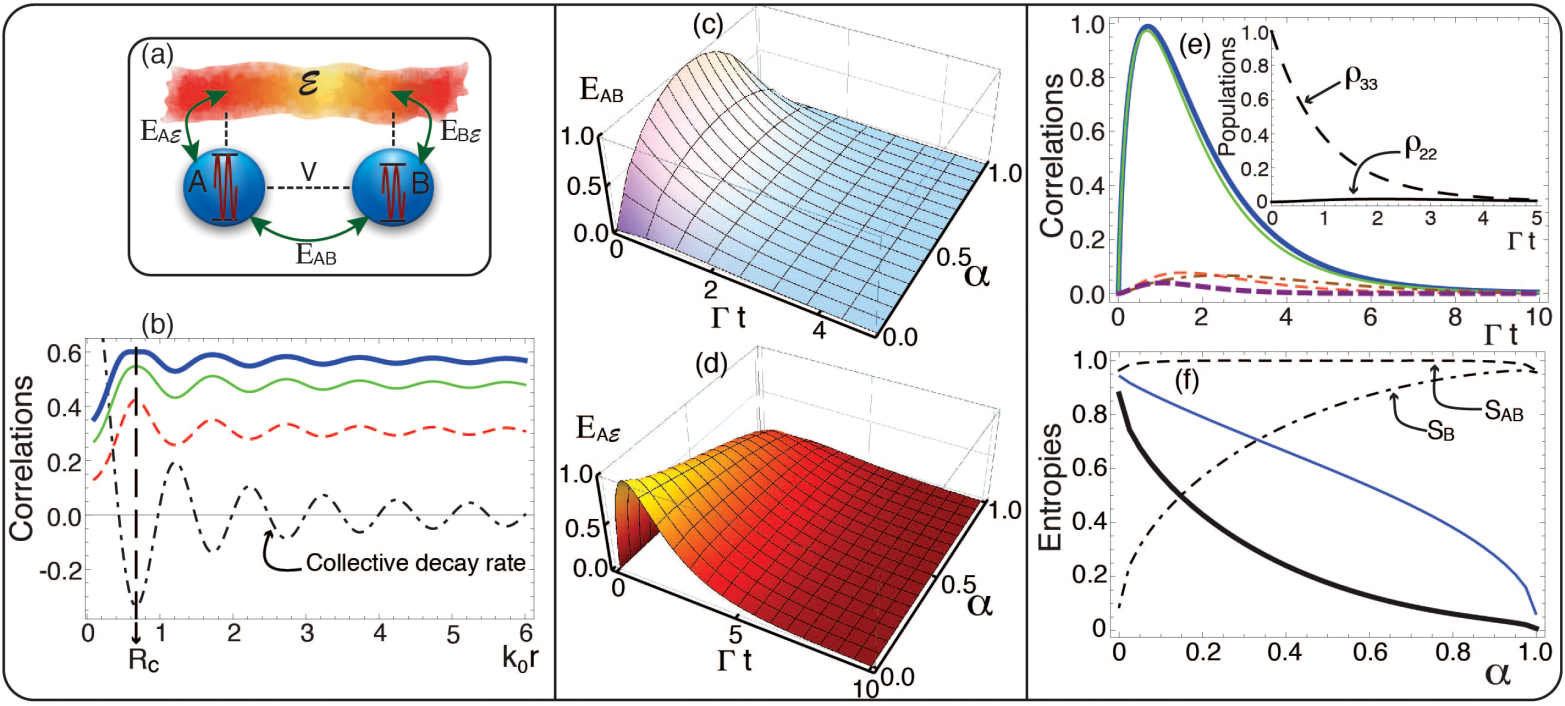
Physical setup, quantum entanglement and correlations in the interacting qubit system. (a) Schematics of the considered 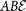 physical system and the flow of quantum information: two two-level emitters (qubits) interacting with a dissipative environment, which are allowed to be optically-driven via external laser excitation. (b) Quantum discord 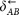 (dashed-red line), 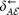 (solid-green line), and entanglement of formation 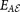 (solid-blue line); initial state |Ψ^+^〉, at *t* = Γ^−1^. The dotted-dashed curve shows the variation in the collective decay rate *γ* due to changes in the interqubit separation *r*. The vertical line signals the optimal 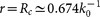. (c) Qubit-qubit (*E_AB_*), and (d) qubit-environment 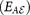 entanglement dynamics for the *α* initial states. (e) Quantum discord 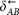 (dashed-red curve), 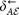 (solid-green curve), and 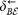 (dotted-dashed-brown curve), and entanglement *E_AB_* (dashed-purple curve), and 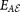 (solid-blue curve). Populations *ρ_ii_* (inset), *α* = 0. (f) Conditional entropy *S_A_*_|B_ (solid-black curve), and 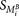 (solid-blue curve) for qubit initial states *α*, *t* = Γ^−1^. *r* = *R_c_*_._

To begin with the quantum dynamics of the qubit-environment correlations, we initially consider resonant qubits, *ω*_1_ = *ω*_2_ ≡ *ω*_0_. In the absence of optical driving, there is an optimal inter-emitter separation *R_c_* which maximises the correlations^43^. In Fig. 1(b) we plot the quantum discord 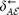, and 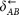, and the entanglement of formation 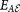 as a function of the interqubit separation *k*_0_*r* at *t* = Γ^−1^. The maximum value reached by each correlation is due to the behaviour of the collective damping *γ*, which reaches its maximum negative value at the optimal separation 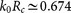, as shown in Fig. 1(b), with *k*_0_ = *ω*_0_/*c*. This is due to the fact that the initial state 
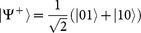
 decays at the rate Γ + *γ* (see equations (8) with *α* = 1/2), and hence the maximum life-time of |Ψ^+^〉 is obtained for the most negative value of *γ*: for any time *t*, the correlations reach their maxima precisely at the interqubit distance *R_c_* (the same result holds for the 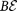 bipartition, not shown).

We stress that it is the collective damping and *not* the dipolar interaction that defines the distance *R_c_*. For a certain family of initial states (which includes |Ψ^+^〉), the free evolution of the emitters is independent of the interqubit interaction *V*^44^: for the initial states 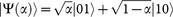, *α* ∈ [0, 1], equation (7) admits an analytical solution and the non-trivial density matrix elements read 
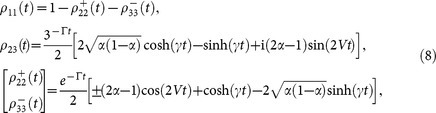
and 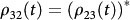. This solution implies that the density matrix dynamics dependence on *V* vanishes for *α* = 1/2 (|Ψ^+^〉), and hence the damping *γ* becomes the only collective parameter responsible for the oscillatory behaviour of the correlations, as shown in Fig. 1(b). A similar analysis can be derived for the initial states 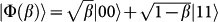. Thus, the ‘detrimental’ behaviour of the system's correlations 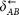 and *E_AB_* reported in [43] is actually explained because such *β* states are not, in general, ‘naturally’ supported by the system's Hamiltonian since they are not eigenstates of 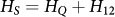.

We now consider the qubits full time evolution and calculate the correlations dynamics for the whole spectrum of initial states |Ψ(*α*)〉, 0 ≤ *α* ≤ 1. The emitters' entanglement *E_AB_* exhibit an asymptotic decay for all *α* values, with the exception of the two limits *α* → 0 and *α* → 1, for which the subsystems begin to correlate with each other and the entanglement increases until it reaches a maximum before decaying monotonically, as shown in Fig. 1(c). The *AB* discord also follows a similar behaviour; this can be seen in Fig. 1(e) for α = 0. Initially, at *t* = 0, the entanglement 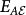 (Fig. 1(d)) equals zero because of the separability of the tripartite 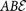 state at such time. After this, the entanglement between *A* and 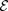 increases to its maximum, which is reached at a different time (*t* ~ Γ^−1^) for each *α*, and then decreases asymptotically. The simulations shown in Figs. 1(c) and (d) have been performed for the optimal inter-emitter separation *R_c_*. These allow to access the dynamical qubit information (entanglement and correlations) exchange between the environment and each subsystem for suitable qubit initialisation.

Figure 1(c) shows the quantum entanglement between identical emitters: *E_AB_* is symmetric with respect to the initialisation *α* = 1/2, i.e., the behaviour of *E_AB_* is the same for the separable states |01〉 and |10〉. In contrast, Fig. 1(d) exhibits a somewhat different behaviour for the entanglement 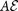, which is not symmetric with respect to *α*: the maximum reached by 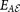 increases as *α* tends to 0 (the discord 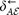 follows the same behaviour–not shown). The dynamical distribution of entanglement between the subsystem *A* and the environment 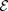 leads to the following: it is possible to have near zero interqubit entanglement (e.g., for the *α* = 1 initialisation) whilst the entanglement between one subsystem and the environment also remains very close to zero throughout the evolution.

This result stresses the sensitivity of the qubit-environment entanglement (and correlations) distribution to its qubit initialisation. To understand why this is so (cf. states |01〉 and |10〉), we analyse the expressions for 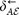 and 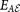. From equations (2) and equations (3), and since *E_AB_* and 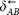 are both symmetric, the asymmetry of 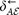 and 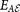 should follow from the conditional entropy *S_A_*_|*B*_ = *S*(*ρ_AB_*) − *S*(*ρ_B_*). This is plotted in the solid-thick-black curve in Fig. 1(f). The behaviour of the conditional entropy is thus reflected in the dynamics of quantum correlations and entanglement between *A* and 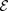, and this can be seen if we compare the behaviour of 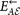 around *t* = Γ^−1^ throughout the *α*-axis in Fig. 1(d), with that of *S_A_*_|*B*_ shown in Fig 1(f). Since this conditional entropy gives the amount of partial information that needs to be transferred from *A* to *B* in order to know *ρ_AB_*, with a prior knowledge of the state of *B*^45^, we have shown that this amount of information may be extracted from the dynamics of the quantum correlations generated between the qubits and their environment.

Interestingly, by replacing the definition of 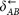
36 into the first equality of equations (2), we find that the entanglement of the 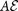 partition is exactly the post-measure conditional entropy of the *AB* partition: 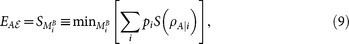
that is, the entanglement between the emitter *A* and its environment is the conditional entropy of *A* after the partition *B* has been measured, and hence the asymmetric behaviour of 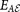 can be verified by plotting this quantity, as shown by the solid-blue curve of Fig. 1(f). A physical reasoning for the asymmetric behaviour of the 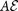 correlations points out that for *α* → 0 the state |10〉 has higher weights throughout the whole dynamics. For instance, for *α* = 0 the subsystem *B* always remains close to its ground state, and transitions between populations *ρ*_22_ and *ρ*_33_ do not take place, as it is shown in the inset of Fig. 1(e). This means that partition *B* keeps almost inactive during this specific evolution and therefore does not share much information, neither quantum nor locally accessible with partition *A* and the environment 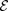. This can be seen from the quantum discord 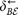, which is plotted as the dotted-dashed-brown curve of Fig. 1(e). We stress that this scenario allows *A* to get strongly correlated with the environment 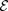.

Although 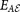 and 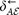 are not ‘symmetric’ with respect to *α*, it is the information flow, i.e., the way the information gets transferred between the qubits and the environment, the quantity that recovers the symmetry exhibited by *E_AB_* in Fig. 1(c). In other words, if the initial state were |01〉, or in general, *α* → 1, the partition *A* would remain almost completely inactive and the flow of information would arise from the bipartite partition 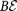 instead of 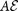. A simple numerical computation for *α* = 0 at *t* = Γ^−1^ shows that 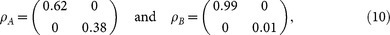
with *S_A_* = 0.96 and *S_B_* = 0.09, respectively. This means that the state of subsystem *B* is close to a pure state (its ground state), and no much information about it may be gained. Instead, almost all the partial information on the state of *A* can be caught regardless of whether the system *B* is measured or not. From this simple reasoning, and by means of the KW relations, the results shown in Figs. 1(c–f) arise. The opposite feature between *ρ_A_* and *ρ_B_* occurs for *α* = 1, and in this case, it is the partition 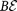 that plays the strongest correlation role.

### Information flow in laser-driven resonant qubits

**Figure 2:**
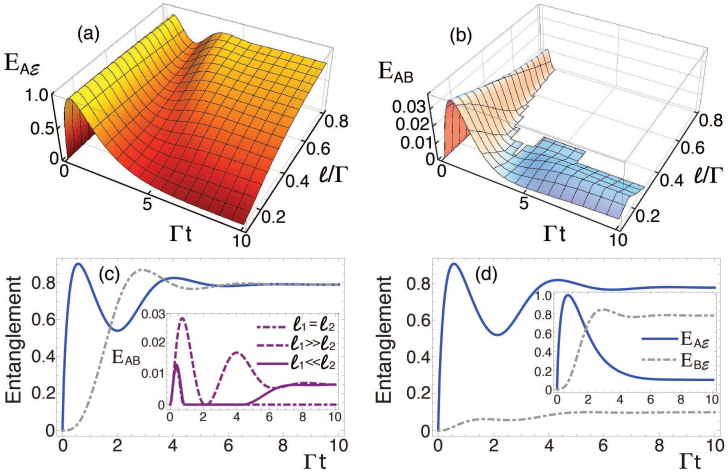
Driven quantum correlations. Dynamics of quantum entanglement (a) 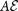 and (b) *AB* as functions of the laser intensity *ℓ*. Main (c) 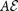 (solid) and 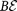 (dotted-dashed), *ℓ*_1_ ≡ *ℓ*_2_ = *ℓ* = 0.8Γ; the inset plots *E_AB_* for three scenarios, main (d) *ℓ*_1_ = 0.8Γ, *ℓ*_2_ = 0; the i nset plots *ℓ*_2_ = 0.8Γ, *ℓ*_1_ = 0. *ρ*_33_(0) = 1, and *r* = *R_c_*.

*H_L_* conveys an additional degree of control of the qubits information (entanglement and discord) flow. Let us consider a continuous laser field acting with the same amplitude, *ℓ*_1_ = *ℓ*_2_ ≡ *ℓ*, on the two emitters, and in resonance with the emitters' transition energy, *ω_L_* = *ω*_0_. The subsystem *A* gets the strongest correlated with the environment for the initial pure state |10〉 in the relevant time regime (see Fig. 1(d)), but this correlation monotonically decays to zero in the steady-state regime. In Fig. 2 we see the effect of the laser driving for the initial state |10〉, for qubits separated by the optimal distance *r* = *R_c_*. The laser field removes the monotonicity in the entanglement and correlations decay between *A* and 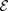, and, as shown in Fig. 2(a), the more intense the laser radiation (even at the weak range *ℓ* ≤ Γ), the more entangled the composite 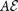 partition becomes. This translates, in turn, into a dynamical mechanism in which the qubit register *AB* gets rapidly disentangled and, even at couplings as weak as *ℓ* ~ 0.4Γ, the qubits exhibit early stage disentanglement, as shown in Fig. 2(b). This regime coincides with the appearance of oscillations in the 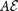 entanglement (see Fig. 2(a)), and steady nonzero 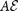 entanglement translates into induced interqubit (*AB*) entanglement suppression by means of the laser field.

By tailoring the laser amplitude we are able to induce and control the way in which the qubits get correlated with each other and with the environment. The graphs 2(c) and (d) show three different scenarios in terms of such amplitude. In graph (c) we plot 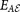 (solid-blue curve) and 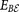 (dashed-dotted-grey curve) for the symmetric light-matter interaction (*ℓ*_1_ = *ℓ*_2_ ≡ *ℓ* = 0.8Γ), which leads to ESD in the partition *AB* (see graph (b)), as well as to a symmetric qubit-environment correlation in the stationary regime. However, as can be seen in main graph (d), where we have assumed *ℓ*_1_ ≫ *ℓ*_2_, the breaking of this symmetry completely modifies the qubit-environment entanglement, and now it is qubit *A* that gets strongly correlated with the environment, while qubit *B* remains weakly correlated during the dynamics. The opposite arises for *ℓ*_1_ ≪ *ℓ*_2_ (inset of panel (d)): 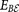 becomes much higher than 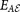, which decays monotonically after reaching its maximum. Remarkably, we notice that these two asymmetric cases lead a nonzero qubit-qubit entanglement as shown in the inset of graph (c), where equal steady entanglement is obtained. It means that the qubits early stage disentanglement^19^,^20^ can be interpreted in terms of the entanglement distribution between the qubits and the environment. We interpret this behaviour as the flow and distribution of entanglement in the different partitions of the whole tripartite system^46^, and hence this result shows that an applied external field may be used to dictate the flow of quantum information within the full tripartite system.

### Flow of information in detuned qubits

We now consider a more general scenario in which each two-level emitter is resonant at a different transition energy, and hence a molecular detuning Δ_−_ = *ω*_1_ − *ω*_2_ arises; *ω*_0_ ≡ (*ω*_1_ + *ω*_2_)/2. Such a detuning substantially modifies the qubit-qubit and qubit-environment correlations. Since Δ_−_ ≠ 0, the *α* = 1/2-time independence of equations (8) with respect to *V* no longer holds, and the critical distance *R_c_* of Fig. 1(b) becomes strongly modified: the information flow exhibits a more involved dynamics precisely at distances *r* < *R_c_*, and the intermediate sub- and super-radiant states are no longer the maximally entangled Bell states.

**Figure 3:**
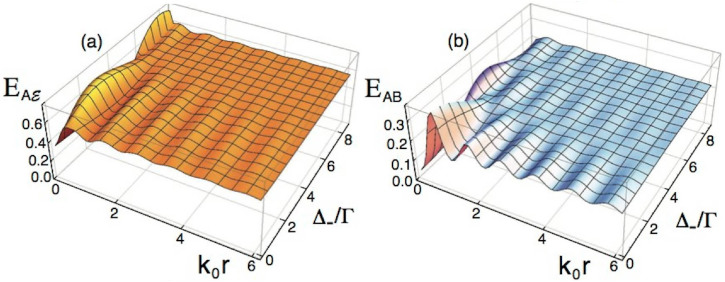
Quantum entanglement for detuned qubits. (a) 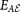, and (b) *E_AB_* dependence on the inter-emitter distance *r* for frequency detuning Δ_−_ ≡ *ω*_1_ − *ω*_2_, and qubits initial state |Ψ^+^〉, *t* = Γ^−1^.

As shown in Fig. 3, the oscillations of 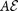 and *AB* entanglement (and their maxima) start to decrease and become flat as the molecular detuning rises (Δ_−_ = 0 corresponds to the case shown in Fig. 1(b)). This means that now, it is not only the collective decay rate *γ* that modulates the behaviour of the entanglement and the correlations, but also the interplay between the detuning Δ_−_ and the dipole-dipole interaction *V*. Note from Fig. 3 that the critical distance *R_c_* for which both the correlations of partition *AB* and those of 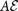 get their maxima disappears with the inclusion of the molecular detuning, and *E_AB_* and 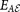 exhibit maxima at different inter-emitter distances as the detuning increases: *E_AB_* remains global maximum for resonant qubits (Δ_−_ = 0) whereas 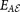 reaches its global maximum for a certain Δ_−_ ≠ 0 (e.g., Δ_−_/Γ = 8 at *k*_0_*r* ~ 0.1), a value for which *E_AB_* is stationary for almost all interqubit separation *r*.

**Figure 4:**
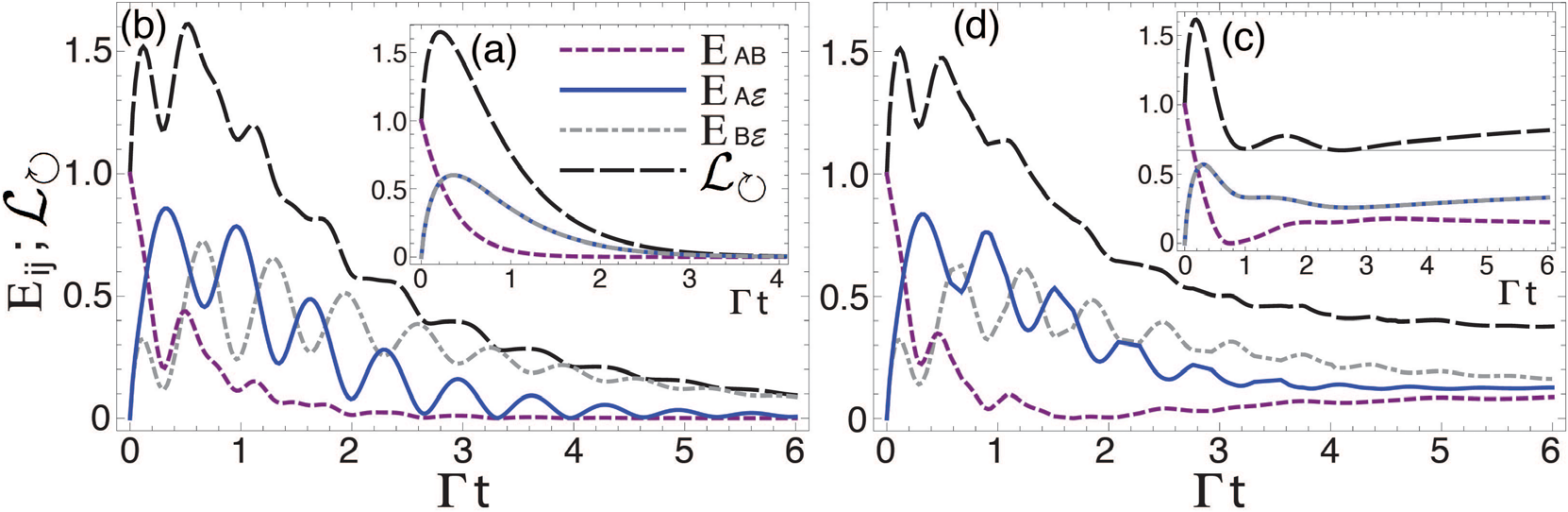
Quantum correlations and local inaccessible information. Entanglement dynamics *E_AB_*, 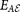, 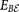, and the flow of quantum information 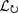. (a) identical, and (b) detuned emitters, Δ_−_ = 8Γ; no laser excitation. *ℓ* = 0.8Γ and *ω*_0_ = *ω_L_*-laser-driven (c) identical, and (d) detuned emitters, Δ_−_ = 8Γ. The initial state |Ψ^+^〉, and *k*_0_*r* = 0.1.

To complete the analysis of the general tripartite 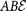 system, we now consider that the asymmetric (detuned) qubits are driven by an external laser field on resonance with the average qubits transition energy, *ω*_0_ = *ω_L_*, as shown in Fig. 4 for the two-qubit initial state |Ψ^+^〉. We have plotted the entanglement dynamics *E_AB_*, 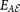, and 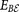. Fig. 4(a) shows the entanglement evolution for two identical emitters in the absence of the external driving. The molecular detuning, and the laser excitation have been included in graphs (b) and (c), respectively. The panel (d) shows the entanglement evolution under detuning and laser driving. The monotonic decay of *E_ij_* for resonant qubits in Fig. 4(a) is in clear contrast with the *E_ij_*-oscillatory behaviour due to the qubit asymmetry, as plotted in Fig. 4(b). A non-zero resonant steady entanglement is obtained thanks to the continuos laser excitation (Fig. 4(c) and (d)). These graphs have been compared with that of the clockwise flow of pairwise locally inaccessible information 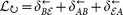
46, as shown in the long-dashed black curve.

## Discussion

We can now interpret the entanglement dynamics of the *AB* partition by means of the dynamics of the clockwise quantum discord distribution in the full tripartite system (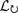), and that of the entanglement of 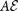 and 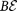 partitions. From the conservation law^27^ between the distribution of the entanglement of formation and discord followed from Eqs. (2) and (3), and noting that 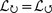 for pure states^46^, where 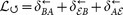, a direct connection between qubit-qubit entanglement and qubit-environment entanglement can be established^46^: 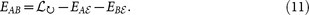


We note from equation (11) and from the pairwise locally inaccessible information that by knowing 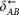 (or 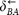), we can exactly compute the qubit-qubit entanglement in terms of the system bipartitions 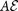 and 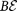. In particular, we show how a profile of the qubit-qubit entanglement might be identified from the partial information obtained from 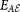 and 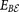, as indicated in the right-handside of equation (11), and shown in Figs. 4(b) and (d) whereby the local minima of *E_AB_* occur at times for which the extrema of 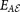 and 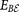 take place. However, it is interesting to note that the locally inaccessible information 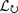, which gives a global information of the whole tripartite system (the distribution of quantum correlations-discord), can be extracted directly from the quantum state of the register. This fact can be demonstrated by replacing equation (9), and its equivalent formula for the bipartition 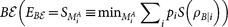, into equation (11):
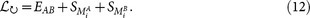


This relation means that the entanglement of partition *AB* plus *local accessible* information of subsystems *A* and *B*, i.e. the post-measured conditional entropies 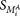 and 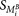, give complete information about the flow of the locally inaccessible (quantum) information.

To summarise, we have shown that the way in which quantum systems correlate or share information can be understood from the dynamics of the register-environment correlations. This has been done via the KW relations established for the entanglement of formation and the quantum discord. Particularly, we have shown that the distribution of entanglement between each qubit and the environment signals the results for both the prior- and post-measure conditional entropy (partial information) shared by the qubits. As a consequence of this link, and in particular equation (9), we have also shown that some information (the distribution of quantum correlations—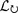) about the whole tripartite system^46^ can be extracted by performing local operations over one of the bipartitions, say *AB*, and by knowing the entanglement of formation in the same subsystem (equation (12)). We stress that these two remarks are completely independent of the considered physical model as they have been deduced from the original definition of the monogamy KW relations (see the Methods section). On the other hand, considering the properties of the specific model here investigated (which may be applicable to atoms, small molecules, and quantum dots arrays), the study of the dynamics of the distribution of qubit-environment correlations led us to establish that qubit energy asymmetry induces entanglement oscillations, and that we can extract partial information about *AB* entanglement by analysing the way in which information (entanglement and discord) flows between each qubit and the environment, for suitable initial states. Particularly, we have shown that the qubits early stage disentanglement may be understood in terms of the laser strength asymmetry which determines the entanglement distribution between the qubits and the environment. In addition, we have also shown that the extrema of the qubit-environment 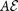 and 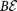 entanglement oscillations exactly match the *AB* entanglement minima. The study here presented has been done without need to explicitly invoque any knowledge about the state of the environment at any time *t* > 0.

An advantage of using the information gained from the system-environment correlations to get information about the reduced system's entanglement dynamics is that new interpretations and understanding of the system dynamics may arise. For instance, one of us^47^ has used this fact to propose an alternative way of detecting the non-Markovianity of an open quantum system by testing the accessible information flow between an ancillary system and the local environment of the apparatus (the open) system.

## Methods

We give a brief introduction to the monogamy relation between the entanglement of formation and the classical correlations established by Koashi and Winter: As a theorem, KW established a trade-off between the entanglement of formation and the classical correlations defined by Henderson and Vedral^37^. They proved that^34^:

### Theorem

*When ρ_AB′_ is B-complement to ρ_AB_,*

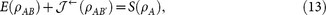
where *B-complement* means that there exist a tripartite pure state *ρ_ABB′_* such that Tr*_B_*[*ρ_ABB′_*] = *ρ_AB′_* and Tr*_B′_*[*ρ_ABB′_*] = *ρ_AB_*, where *S_A_* : = *S*(*ρ_A_*) is the von Neumann entropy of the density matrix *ρ_A_* ≡ Tr*_B_*[*ρ_AB_*] = Tr*_B′_*[*ρ_AB′_*], *E_AB_* : = *E*(*ρ_AB_*) is the entanglement of formation, and 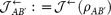 leads the classical correlations.

For our purpose we only show some steps in the proof of the KW relation (equation (13)); the complete proof can be straight-forwardly followed in^34^. By starting with the definition of the entanglement of formation: 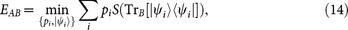
where the minimum is over the ensamble of pure states {*p_i_*, |*ψ_i_*〉} satisfying 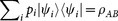, it is possible to show, after some algebra, that 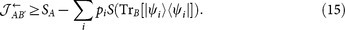
Conversely, from the definition of classical correlations^37^: 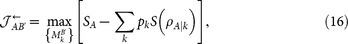
where 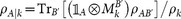 is the state of party *A* after the set of measurements 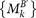 has been done on party *B′* with probability 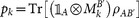. Let us assume that 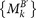 is the set achieving the maximum in equation (16) such that one can write 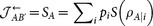, as the operators 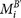 may be of rank larger than one, by decomposing them into rank-1 nonnegative operators such that 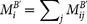, one can show the following 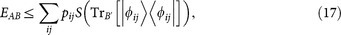
where {*p_ij_*, |*φ_ij_*〉} is an ensamble of pure states with 
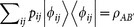
 as the set of measurements 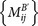 is applied to the pure tripartite state *ρ_ABB′_*. The relationship between the sets 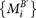 and 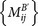 is through: 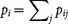 and 
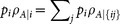
.

Noting that 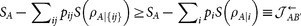, due to the concavity of the von Neumann entropy, but that the opposite inequality arises by the own definition of the classical correlations, one concludes that 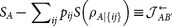. Then, by putting together the results of equations (15) and (16), one achieves the equation (13).

By introducing the definition of quantum discord^36^: 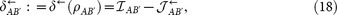
where 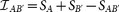 is the quantum mutual information of the bipartition *AB*′, into equation (13), one gets 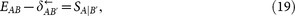
with *S_A_*_|*B*′_ = *S_AB′_* − *S_B′_* the conditional entropy. Noting that *S_A_*_|*B′*_ = −*S_A_*_|*B*_ because the tripartite state *ρ_ABB′_* is pure, and changing the subscript *B′* to 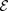, equation (19) gives rise to the expression for 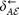 in equation (3). The rest of equalities in equations (2) and (3) are obtained by moving the three subscripts and applying the corresponding classical correlations to the appropriate bipartition.
